# COVID-19-associated hypertriglyceridemia and impact of treatment

**DOI:** 10.3389/fmed.2024.1326156

**Published:** 2024-02-21

**Authors:** Rasha Kaddoura, Mohamed Izham Mohamed Ibrahim, Maha Al-Amri, Arun Prabhakaran Nair, Ahmad Alharafsheh, Sumaya Alsaadi Alyafei, Mutaz Albakri

**Affiliations:** ^1^Pharmacy Department, Heart Hospital, Hamad Medical Corporation, Doha, Qatar; ^2^College of Pharmacy, QU Health, Qatar University, Doha, Qatar; ^3^Department of Infectious Disease, Communicable Disease Center, Hamad Medical Corporation, Doha, Qatar; ^4^Pharmacy Department, Hazm Mebaireek General Hospital, Hamad Medical Corporation, Doha, Qatar; ^5^Pulmonary Medicine Department, Hamad General Hospital, Hamad Medical Corporation, Doha, Qatar

**Keywords:** COVID-19, fenofibrate, hypertriglyceridemia, omega-3, SARS-CoV-2, triglycerides

## Abstract

**Background:**

Coronavirus disease 2019 (COVID-19) associated hypertriglyceridemia was observed among patients admitted to intensive care units (ICU) in Qatar. This study aimed to describe COVID-19-associated-hypertriglyceridemia in ICU patients and the impact of treating hypertriglyceridemia on clinical outcomes.

**Methods:**

A retrospective observational cohort study of adult patients who were admitted to the ICU with a confirmed diagnosis of COVID-19 pneumonia according to the World Health Organization criteria. Hypertriglyceridemia was defined as triglyceride level of 1.7 mmol/L (≥150 mg/dL) and severe hypertriglyceridemia as fasting TG of ≥5.6 mmol/L (≥500 mg/dL).

**Results:**

Of 1,234 enrolled patients, 1,016 (82.3%) had hypertriglyceridemia. Median age was 50 years and 87.9% were males. Patients with hypertriglyceridemia showed significantly longer time to COVID-19 recovery, ICU and hospital stay, and time to death (29.3 vs. 16.9 days) without a difference in mortality between groups. Of patients with hypertriglyceridemia, 343 (33.8%) received treatment (i.e., fibrate and/or omega-3). Patients in treatment group showed longer time to COVID-19 recovery and hospital stay with no difference in death rates in comparison with those in no-treatment group. Relatively older patients were less likely to experience hypertriglyceridemia (odd ratio (OR) 0.976; 95% CI: 0.956, 0.995) or to receive treatment (OR 0.977; 95% CI: 0.960, 0.994). Whereas patients who received tocilizumab were more likely to experience high TG level (OR 3.508; 95% CI: 2.046, 6.015) and to receive treatment for it (OR 2.528; 95% CI: 1.628, 3.926).

**Conclusion:**

Hypertriglyceridemia associated with COVID-19 did not increase death rate, but prolonged time to death and length of stay. Treating hypertriglyceridemia did not translate into improvement in clinical outcomes including mortality.

## 1 Introduction

Coronavirus disease 2019 (COVID-19) that is caused by Severe Acute Respiratory Syndrome Coronavirus-2 (SARS-CoV-2) was announced a pandemic on March 11, 2020, by the World Health Organization (WHO) (1). The pandemic has infected over 164 million people globally causing three million deaths by May 2021 (2). In addition, it has significantly increased hospitalization due to COVID-19 pneumonia with other organ disease (3). The disease has usually a self-limited course, (2) but moderate or severe symptoms were reported in approximately 20% of COVID-19 patients, with a progression rate to critical stages of 5% (4). SARS-CoV-2 is primarily a respiratory illness, but manifestations vary widely, affecting multiple extrapulmonary systems, (5) including hepatic, gastrointestinal, cardiac, renal, and even multi-organ damage (4). Thus, resulting in management dilemmas (5).

Hypertriglyceridemia associated with SARS-CoV-2 has been reported earlier during the start of the pandemic. There was a case report showing severe acute pancreatitis in association with hypertriglyceridemia [i.e., 48 mmol/L (4,247 mg/dL)] and COVID-19 infection (5). Another study found that triglycerides (TG) levels in COVID-19 patients were significantly higher than the levels in normal subject (*p* < 0.01), but significantly decreased in critical cases as compared with mild and severe cases (*p* < 0.01) (6). A retrospective study on hospitalized COVID-19 patients showed that the pro-inflammatory state caused by the SARS-CoV-2 infection may exert an intense effect on lipid metabolism leading to higher TG levels (7). In severe SARS-CoV-2 pneumonia there are alterations in fatty acid profile, although such alterations are part of the host defense system, they favor viral replication (8).

Severe hypertriglyceridemia can have rapid onset in critical COVID-19 cases (9). Although hyperlipidemia can mediate pancreatic injury and acute pancreatitis, only small proportion of patients with COVID-19 presented with acute pancreatitis (2). Rapid lowering of serum TG levels in severe hypertriglyceridemia is paramount to prevent development of acute pancreatitis especially when a patient’s TG level is very high, i.e., typically >10 mmol/L (884.96 mg/dL) (10). Hypertriglyceridemia has been observed in COVID-19 patients admitted to intensive care units (ICU) in Qatar. The TG levels have been measured regularly in these patients. Furthermore, the treatment of the COVID-19-associated hypertriglyceridemia was prescribed at the ICU physician’s discretion. There has been no report published to examine similar observation. This study was hoped to give a better understanding about hypertriglyceridemia associated with COVID-19. Herein, this study describes hypertriglyceridemia in COVID-19 patients admitted to ICU and the impact of treating hypertriglyceridemia on patient’s clinical outcomes.

## 2 Methods

The research question was addressed through a retrospective observational cohort study of COVID-19 patients who were admitted to the ICU in Qatar between February 1, 2020 and August 23, 2020. The ICU were from five hospitals (Hazm Mebaireek General Hospital, The Cuban Hospital, Hamad General Hospital, Al-Khor Hospital, Al-Wakra Hospital), under Hamad Medical Corporation (HMC), that were considered COVID-19 facilities at the time of the pandemic. The study was approved by the Institutional Review Board of the Medical Research Center (MRC-01-20-834) and a waiver of informed consent was granted given the retrospective nature of the study. The data has been de-identified before being shared with the investigators. The study was conducted in full conformance with principles of the Declaration of Helsinki, Good Clinical Practice and within the laws and regulations of the Ministry of Public Health in Qatar.

A list of patients who were admitted to ICU in the specified period was generated from electronic medical records. A cross-sectional review was performed for eligible patients and the relevant pre-specified data was collected, including patient demographics, comorbidities, test results, in-hospital treatment, and pre-specified outcomes. Patients were included if they were adults (≥18 years) admitted to ICU with a diagnosis of COVID-19 pneumonia and confirmed SARS-CoV-2 infection. Exclusion criteria were patients who had incomplete key data, mild COVID-19 disease as per WHO severity criteria and with elevated TG level [1.7 mmol/L (>150 mg/dL)] prior to COVID-19 diagnosis. In addition, pregnant or lactating women, and patients on lipid emulsion as a component of total parenteral nutrition were excluded.

During the recruitment period, confirmed SARS-CoV-2 infection was determined by qualitative reverse transcriptase polymerase chain reaction (RT-PCR), or other commercial or public health assay in any specimen during the current hospital admission. RT-PCR may be from throat swab and/or sputum and/or lower respiratory tract samples. Symptomatic COVID-19 illness was confirmed by radiographic infiltrates by imaging, e.g., chest X-ray, computed tomography, etc. COVID-19 pneumonia was defined according to the WHO criteria for severe and critical pneumonia including acute respiratory distress syndrome (ARDS), and sepsis (11). Hypertriglyceridemia at the time of ICU admission was defined as TG level of 1.7 mmol/L (≥150 mg/dL), moderate hypertriglyceridemia as fasting or non-fasting TG of 2.0–5.6 mmol/L (175–499 mg/dL), and severe hypertriglyceridemia as fasting TG of ≥5.6 mmol/L (≥500 mg/dL) (12).

### 2.1 Statistical analysis

A two-tier analysis was performed, the first tier examined the predictors associated with the elevated TG levels, and whether such elevations impacted clinical outcomes in all patients. The second tier involved the patients with high TG only and compared patient characteristics and clinical outcomes between those who received treatment (fenofibrate, omega 3) and those who did not. Data was managed using Excel and SPSS vs. 29 (IBM Corp. Released 2021. IBM SPSS Statistics for Windows, Version 29.0. Armonk, NY: IBM Corp) programs. Normality test, Kolmogorov–Smirnov was carried out to test for data normality. Continuous variables were presented as mean (standard deviation, SD) or median (interquartile range). Categorical variables were expressed as frequencies and percentages. Inferential statistics, Student’s *t*-test, or Mann–Whitney test was used to compare between the groups, while categorical variables were compared between the groups using Chi-square and Fisher’s exact tests. Multiple logistic regression analyses were performed to examine the association between (1) the levels of TG (high versus low) and (2) receiving hypertriglyceridemia treatment or not with various predictor variables which were significantly associated with the outcome variable. Assumptions of logistic regression, such as linearity of log-odds and absence of multicollinearity, were checked. Coefficients and odds ratios were calculated for each predictor variable in the models. Wald tests were used to assess the statistical significance of individual coefficients. The priori alpha level was set at 0.05.

## 3 Results

### 3.1 Hypertriglyceridemia versus none

#### 3.1.1 Baseline characteristics

Of 1,234 patients who were admitted to ICU with a positive SARS-CoV-2 and ARDS, 1,016 (82.3%) patients had high TG levels. Their median age was 50 years and 87.9% were males. The most common comorbidities were diabetes (59.2%), hypertension (52.2%), and renal impairment (14.1%) (Table 1).

**Table 1:** Baseline characteristics based on triglycerides levels.

Variable	High triglycerides level		*p* value
	Yes (*n*, %) *N* = 1,016 (82.3%)	No (*n*, %) *N* = 218 (17.7%)	
Male	893 (87.9)	172 (78.9)	<0.001
Female	123 (12.1)	46 (21.1)	
*Age group (year)*			
≤20	2 (02.)	1 (0.5)	0.003
21–30	44 (4.3)	10 (4.6)	
31–40	187 (18.4)	34 (15.6)	
41–50	301 (29.6)	43 (19.7)	
51–60	269 (26.5)	59 (27.1)	
>60	213 (21.0)	71 (32.6)	
Qatari	72 (7.1)	35 (16.1)	<0.001
Non-Qatari	944 (92.6)	183 (83.9)	
Age (year), median (Δ IQR)	50.0 (18)	53.0 (20.0)	<0.001
BMI (kg/m^2^), median (Δ IQR)	27.8 (6.7)	27.3 (7.0)	0.041
*Smoking and alcohol status*			
Non-smoker	562 (55.3)	133 (61.0)	0.013
Smoker	47 (4.6)	16 (7.3)	
Ex-smoker	54 (5.3)	16 (7.3)	
Unknown	353 (34.7)	53 (24.3)	
Non-alcoholic	518 (51.0)	130 (59.6)	0.019
Alcoholic	39 (3.8)	12 (5.5)	
Former	23 (2.3)	1 (0.5)	
Unknown	436 (42.9)	75 (34.4)	
*Comorbidities*			
Hypertension	530 (52.2)	138 (63.3)	0.003
Diabetes mellitus	601 (59.2)	131 (60.1)	0.798
CAD	88 (8.7)	49 (22.5)	<0.001
PVD	5 (0.5)	2 (0.9)	0.448
History of DVT/PE	61 (6.0)	11 (5.0)	0.584
Heart Failure	55 (5.4)	32 (14.7)	<0.001
Atrial fibrillation	89 (8.8)	23 (10.6)	0.404
CKD/ESRD	143 (14.1)	32 (14.7)	0.817
Liver disease	56 (5.5)	20 (9.2)	0.041
COPD	25 (2.5)	6 (2.8)	0.803
ILD	5 (0.5)	0 (0.0)	0.299
PUD	12 (1.2)	7 (3.2)	0.027
Solid tumor	27 (2.7)	9 (4.1)	0.242
Lymphoma	7 (0.7)	1 (0.5)	0.701
Leukemia	10 (1.0)	0 (0.0)	0.141
Organ transplant	16 (1.6)	2 (0.9)	0.463
Dementia	13 (1.3)	4 (1.8)	0.523

Chi-square, Fisher Exact’s, and Mann–Whitney tests were used at alpha 0.05. CAD, coronary artery disease; CKD, chronic kidney disease; COPD, chronic obstructive pulmonary disease; DVT, deep vein thrombosis; ESRD, end-stage renal disease; ILD, interstitial lung disease; IQR, interquartile range; PE, pulmonary embolism; PUD, peptic ulcer disease; PVD, peripheral vascular disease.

#### 3.1.2 Vital signs and blood tests

**Figure 1:**
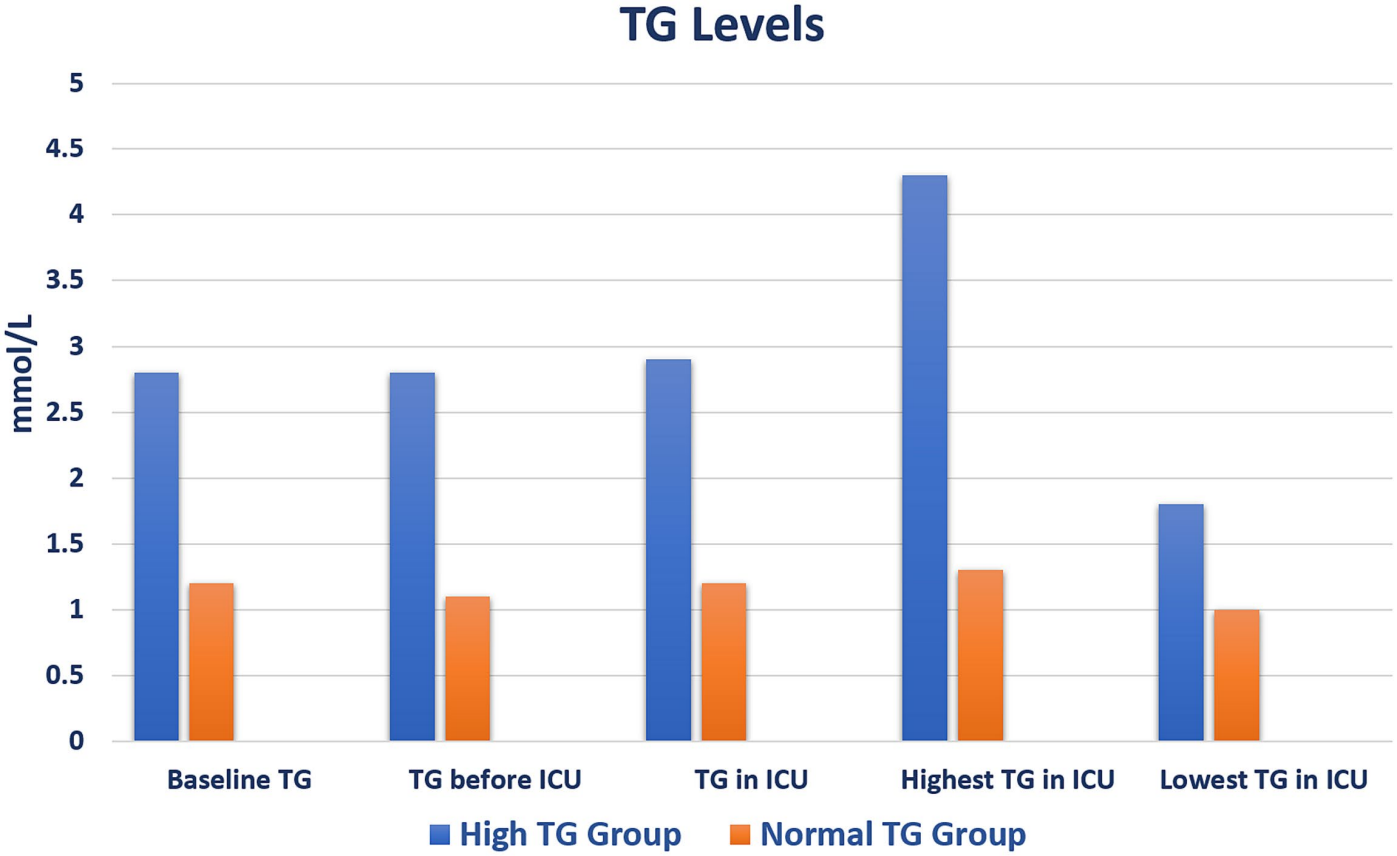
Triglyceride level values during admission. ICU, intensive care unit; TG, triglyceride.

Upon ICU admission, patients with high TG levels had higher heart and respiratory rates with lower oxygen saturation than patients without TG elevation. Blood levels of glucose, lactate dehydrogenase, C-reactive protein (C-RP), procalcitonin, ferritin, interleukin (IL)-6 were higher in patients with hypertriglyceridemia (Supplementary Table S1). Their highest and lowest mean TG level were 4.3 ± 2.2 and 1.8 ± 0.9 mmol/L, respectively (Figure 1; Supplementary Table S2).

#### 3.1.3 Medications and oxygen therapy

Home medications that may affect TG level did not differ between the groups. Only a small number of patients received fibrate therapy before hospital admission. The proportion of patients who received intravenous insulin, broad-spectrum antibiotics, antifungal agents, and COVID-19 therapy was higher in the hypertriglyceridemia group than the comparator. Patients with hypertriglyceridemia required more invasive and non-invasive oxygen therapy (98.1% vs. 91.3%, *p* < 0.001) and for longer period (18.6 vs. 11.3 days, *p* < 0.001) compared with the other group (Table 2).

**Table 2:** At-home and in-hospital medications based on triglycerides levels.

Variable	High triglycerides levels		*p* value
	Yes (*n*, %); *N* = 1,016	No (*n*, %); *N* = 218	
*Pre-specified medications at home*			
Olanzapine	4 (0.4)	3 (1.4)	0.080
Cyclosporine	4 (0.4)	0 (0)	0.353
Fenofibrate	20 (2.0)	3 (1.4)	0.557
Gemfibrozil	3 (0.3)	0 (0)	1.000
Methylprednisolone	47 (4.6)	7 (3.2)	0.354
Prednisolone	72 (7.1)	23 (10.6)	0.082
Tacrolimus	8 (0.8)	2 (0.9)	0.693
*Hypertriglyceridemia treatment in hospital*			
Gemfibrozil	8 (0.78)	–	
Omega-3	125 (12.3)	–	
Fenofibrate	253 (24.9)	–	
*Medications in hospital*			
Insulin	913 (89.9)	182 (83.5)	0.026
Olanzapine	6 (0.6)	1 (0.5)	0.070
Atorvastatin	192 (18.9)	76 (34.9)	<0.001
Pravastatin	3 (0.3)	0 (0.0)	0.053
Rosuvastatin	81 (8.0)	25 (11.5)	0.012
Simvastatin	6 (0.6)	1 (0.5)	0.070
Any statin	260 (27.5)	93 (48.4)	<0.001
Beta-lactams^*^	941 (92.6)	175 (80.3)	<0.001
Meropenem/piperacillin-tazobactam	757 (74.5)	117 (53.7)	<0.001
Antifungal agents^&^	272 (26.8)	29 (13.3)	<0.001
Hydroxychloroquine	835 (82.2)	126 (57.8)	<0.001
Azithromycin	947 (93.2)	178 (81.7)	<0.001
Methylprednisolone/dexamethasone	926 (91.1)	140 (64.2)	<0.001
Tocilizumab	616 (60.6)	37 (17.0)	<0.001
Interferon	140 (13.8)	19 (8.7)	0.043
IVIG use	214 (21.2)	26 (11.9)	0.002
Oseltamivir	947 (93.2)	178 (81.7)	<0.001
Lopinavir/ritonavir	562 (55.3)	53 (24.3)	<0.001
Favipiravir	174 (17.1)	63 (28.9)	<0.001
Darunavir	43 (4.2)	3 (1.4)	0.043
Ribavirin	121 (11.9)	7 (3.2)	<0.001
VTE prophylaxis^#^	1,008 (99.2)	213 (97.7)	0.063
*Oxygen therapy*			
Non-invasive O_2_ therapy	996 (98.0)	199 (91.3)	<0.001
Invasive O_2_ therapy	659 (64.9)	73 (33.5)	<0.001
All O_2_ Therapy	997 (98.1)	199 (91.3)	<0.001
	**Median (Δ IQR)**	**Median (Δ IQR)**	
Duration of non-invasive O_2_ (day)	14.2 (15.2)	11.3 (15.9)	<0.001
Duration of invasive O_2_ (day)	3.9 (12.8)	0.0 (2.8)	<0.001
Duration of all O_2_ (Day)	18.6 (24.1)	11.3 (18.5)	<0.001

Chi-square test and Fisher Exact’s test were used at alpha 0.05. ^*^Ceftriaxone, co-amoxiclav, cefepime, ceftazidime. ^&^Anidulafungin, caspofungin, amphotericin. ^#^Heparin, enoxaparin, fondaparinux, or dalteparin. IQR, interquartile range; IVIG, intravenous immunoglobulin; O2, oxygen; VTE, venous thromboembolism.

#### 3.1.4 Clinical outcomes and predictors

**Figure 2:**
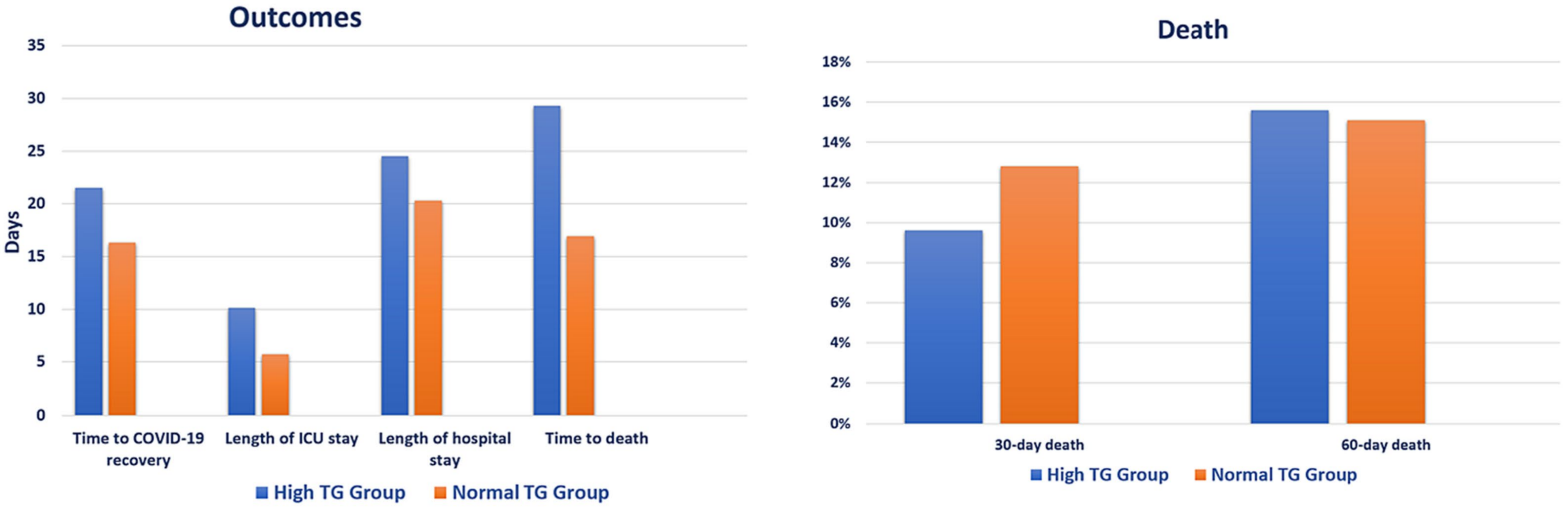
Clinical outcomes based on triglycerides levels. ICU, intensive care unit; TG, triglyceride.

Patients in the hypertriglyceridemia group showed longer time to COVID-19 recovery (21.5 vs. 16.3 days, *p* = 0.003), ICU stay (10.1 vs. 5.7 days, *p* < 0.001), hospital stay (24.5 vs. 20.3 days, *p* < 0.001), and time to death (29.3 vs. 16.9 days, *p* < 0.001) compared with patients in the comparison group. However, there was no difference in 30-day (9.6% vs. 12.8%, *p* = 0.157) and 60-day (15.6% vs. 15.1%, *p* = 0.850) death between the groups (Figure 2; Supplementary Table S3). Relatively older patients were less likely to experience high TG level [odd ratio (OR) 0.976; 95% CI: 0.956, 0.995, *p* = 0.016], whereas patients who received antifungal and tocilizumab were more likely to experience high TG level [(OR 2.381; 95% CI: 1.296, 4.374, *p* = 0.005) and (OR 3.508; 95% CI: 2.046, 6.015, *p* < 0.001), respectively] (Table 3).

**Table 3:** Predictors of high triglycerides levels.

Variable	OR (95% CI)	Value of *p*
Sex	1.283 (0.687, 2.396)	0.434
Age	0.976 (0.956, 0.995)	0.016
CAD	0.763 (0.345, 1.690)	0.506
HF	0.623 (0.268, 1.450)	0.272
Procalcitonin	0.989 (0.964, 1.015)	0.397
Ferritin	1.000 (1.000, 1.000)	0.051
IL-6	1.000 (1.000, 1.000)	0.913
Antifungal agents	2.381 (1.296, 4.374)	0.005
Methylprednisolone/dexamethasone	2.141 (0.933, 4.913)	0.072
Tocilizumab	3.508 (2.046, 6.015)	<0.001
Meropenem/piperacillin-tazobactam	0.937 (0.509, 1.726)	0.835

CAD, coronary artery disease; CI, confidence interval; HF, heart failure; IL-6, interleukin-6; OR, odds ratio.

### 3.2 Treatment of hypertriglyceridemia versus none

Of the 1,016 (82.3%) patients with hypertriglyceridemia, 343 (33.8%) received treatment. Baseline characteristics, vital signs, blood tests, TG levels of patients who received and did not receive treatment are presented in Supplementary Tables S4–S6. Fenofibrate was the most frequently prescribed agent (73.8%) in hospital, followed by omega-3 (36.4%). The proportion of patients who received broad-spectrum antibiotics, antifungal agents, COVID-19 therapy, and invasive oxygen was higher in the hypertriglyceridemia treatment group than the comparator. Patients in the no-treatment group received more statin therapy than in the treatment group (27.6% vs. 21.8%, *p* = 0.015; Supplementary Table S7).

**Figure 3:**
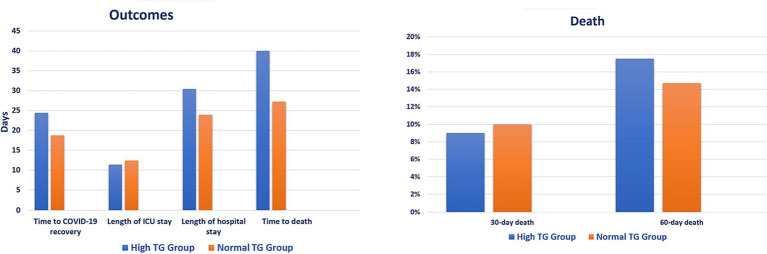
Clinical outcomes based on hypertriglyceridemia treatment. ICU, intensive care unit; TG, triglyceride.

Patients in the hypertriglyceridemia treatment group showed longer time to COVID-19 recovery (24.4 vs. 18.7 days, *p* < 0.001), hospital stay (30.4 vs. 23.9 days, *p* < 0.001), and time to death (40.0 vs. 27.2 days, *p* = 0.020) compared with patients in the no-treatment group. However, there was no difference in 30-day (9.0% vs. 10.0%, *p* = 0.639) and 60-day (17.5% vs. 14.7%, *p* = 0.248) death between the groups (Figure 3; Supplementary Table S8). Patients of younger ages and who received interferon are less likely to receive treatment for hypertriglyceridemia [(OR 0.977; 95% CI: 0.960, 0.994, *p* = 0.008) and (OR 0.402; 95% CI: 0.203, 0.79, *p* = 0.009), respectively], whereas patients who received tocilizumab are more likely to be treated for hypertriglyceridemia (OR 2.528; 95% CI: 1.628, 3.926, *p* < 0.001) (Table 4).

**Table 4:** Predictors of receiving hypertriglyceridemia treatment.

Variable	OR (95% CI)	Value of *p*
Age	0.977 (0.960, 0.994)	0.008
Hypertension	0.819 (0.525, 1.277)	0.377
C-RP	1.000 (0.999, 1.002)	0.657
IL-6	1.000	0.734
Insulin	1.352 (0.360, 5.082)	0.655
Any statin	0.712 (0.436, 1.161)	0.173
Antifungal agents	1.541 (0.926, 2.564)	0.096
Meropenem/piperacillin-tazobactam	1.093 (0.640.869)	0.744
Methylprednisolone/dexamethasone	1.390 (0.564, 3.426)	0.474
Tocilizumab	2.528 (1.628, 3.926)	<0.001
Interferon	0.402 (0.203, 0.799)	0.009
IVIG	1.129 (0.667, 1.911)	0.651

CI, confidence interval; C-RP, C-reactive protein; IL-6, interleukin-6; IVIG, intravenous immunoglobulin; OR, odds ratio.

## 4 Discussion

This retrospective study described hypertriglyceridemia in COVID-19 patients admitted to the ICU and the impact of treating hypertriglyceridemia on patient’s clinical outcomes. Patients with hypertriglyceridemia had higher inflammatory markers levels, and more patients received broad-spectrum antibiotics, antifungal agents, and COVID-19 treatment, and oxygen therapy than patients without hypertriglyceridemia. Although the length of ICU and hospital stay was longer in patients with high TG levels, mortality did not differ between the two groups. Treating hypertriglyceridemia in COVID-19 patients did not reduce the risk of mortality. To put the findings of our study in the context of the published reports, several studies have reported an increased rate of hypertriglyceridemia in COVID-19 patients. However, we have not identified any study that reported the impact of treating hypertriglyceridemia.

A retrospective analysis of clinical reports of Italian patients hospitalized for COVID-19 provided, for the first time, evidence on the association between atherogenic dyslipidemia on hospital admission, especially hypertriglyceridemia, and critical disease course in hospital, defined as in-hospital death or endotracheal intubation. Critical COVID-19 cases presented with increased levels TG, fasting glucose, procalcitonin, C-RP, D-dimer, and IL-6 (*p* < 0.01 for all) and lower levels of high-density lipoprotein (*p* = 0.003) compared with noncritical cases. Atherogenic dyslipidemia was correlated to in-hospital mortality (OR 3.63; 95% CI: 1.24–5.77, *p* < 0.009) and composite of mortality and intubation (OR 2.53; 95% CI: 1.16–6.32, *p* = 0.018). In a linear regression, TG levels were positively associated with procalcitonin, C-PR and D-dimer (*p* < 0.05 for all), which was associated with worse COVID-19 prognosis [(OR 2.62; 95% CI: 1.26–4.13, *p* < 0.005; for TG levels) and (OR 4.03; 95% CI: 1.55–8.22, *p* < 0.012; for C-RP levels)]. The investigators encouraged evaluating lipid profile alongside known risk factors in patients presenting with severe COVID-19 (13). Moreover, a study used Mendelian randomization in European population from United Kingdom Biobank to examine the lipid-related traits causal effects on the risk of COVID-19, suggested that higher TG serum level was linked to greater COVID-19 susceptibility (odds ratio (OR) per standard deviation increase in lifelong TG levels, 1.065; 95% CI, 1.001–1.13, *p* = 0.045), COVID-19 severity (OR, 1.274; 95% CI, 1.08–1.50, *p* = 0.004), and hospitalization (OR, 1.174; 95% CI, 1.04–1.33, *p* = 0.012). However, the mechanism that explains the association of hypertriglyceridemia with worsening COVID-19 course is unclear (14).

The proposed mechanism for the occurrence of hypertriglyceridemia seems multifaceted (15, 16). Infection triggers immunological response that dysregulates lipid metabolism (15). Lipoproteins play a role in innate immunity and variations in their levels have been observed in a variety of inflammatory disorders (17). Hypertriglyceridemia characterizes lipoprotein metabolism changes during an inflammatory process (15). High TG concentrations can result from an increased rate of very-low-density lipoprotein (VLDL) secretion from the liver, from a decreased rate of removal of VLDL and TG, or from a combination of changes in both processes. Tumour necrosis factor (TNF)-α has also been shown to elevate serum TG level by increasing the hepatic production and decreasing lipoprotein lipase (LPL) activity in adipose tissue (18–21). Infection severity is inversely proportional to the VLDL elimination due to reduced apolipoprotein E and lipoprotein lipase. Lipoprotein lipase activity inhibitor can inhibit the metabolism of TG-rich lipoproteins, hence playing a role in lipid metabolism and contributing to pro-atherogenic alterations during severe inflammation (15). Other immunological aspects in severe cases that contribute to elevated TG levels include increased levels of inflammatory markers such as IL-2, IL-7, IL-10, TNF, macrophage inflammatory protein, and granulocytes colony-stimulating factor (15). Furthermore, it has been suggested that elevated TG levels are due to increased adipose tissue lipolysis in patients with COVID-19 (8), and possible increased expression of angiotensin-converting enzyme 2 (ACE2) protein in SARA-CoV-2 (15). Rapid severe hypertriglyceridemia onset in COVID-19 patients has been observed secondary to acute liver impairment, medications, or hemophagocytic lymphohystiocytosis syndrome, medication-induced, or acute liver failure (9). Hypertriglyceridemia, as a result, can provoke a new systemic inflammation and potentiating a pre-existing one because it is considered an activator of the NLRP3 inflammasome (15).

Several retrospective studies have investigated drug-induced hypertriglyceridemia (22) in hospitalized COVID-19 patients such as tocilizumab use (22). Tocilizumab, an IL-6 receptor antibody, is one of the treatment options in severe COVID-19. Chronic use of tocilizumab in other indications has led to elevated TG levels and acute pancreatitis. There is limited evidence about the short-term tocilizumab and the occurrence of hypertriglyceridemia in COVID-19 patients. Two reported COVID-19 patients were found to develop acute hypertriglyceridemia with tocilizumab use, one with acute pancreatitis and the other one without it. The relationship between tocilizumab and hypertriglyceridemia was assessed using the Naranjo Probability Scale which yielded a score of 7, i.e., probable relationship (22). In our study, tocilizumab predicted both hypertriglyceridemia and the consequent treatment. Non-medication COVID-19-associated acute pancreatitis has been reported in an incidence of 0.70% and has been associated with worse prognosis such as higher mortality, longer hospital stay, more ICU admission, and persistent organ failure (2). However, there is not conclusive evidence to consider COVID-19 as an independent factor for acute pancreatitis (2, 23). The potential mechanism of pancreatitis in COVID-19 patients is presumably the detrimental effects of hypertriglyceridemia still exist, which might be a mediating factor in the pathogenesis of pancreatic injury and acute pancreatitis (2, 23). Transient inhibition of lipoprotein lipase due to COVID-19 infection leads to elevated TG. The subsequent hydrolysis of excessive TG by pancreatic lipase and excessive formation of free fatty acids would cause inflammation and pancreatic injury (23). Acquired LPL inhibition, as a rare complication of COVID-19, due to overproduction of autoantibodies may increase the incidence of pancreatitis by 360-fold, with poor prognosis once pancreatitis is present. The diagnosis of acquired LPL deficiency should be considered once severe hypertriglyceridemia develops following COVID-19 and vice versa (16). In our study, we could not identify the cases of acute pancreatitis due to limitations in the data collection process.

Serum TG level may be an important factor influencing recovery from COVID-19, therefore lowering serum levels may promote recovery (15). Continuous insulin infusion has been reported to provide rapid TG levels lowering in mechanically ventilated COVID-19 patients (9). There is potential benefit of plasmapheresis to remove proinflammatory cytokines and toxins that trigger cytokine storm syndrome which is suggested to be associated hypertriglyceridemia. It has been suggested to target normal TG levels when using plasmapheresis to prevent cytokine storm (15). Fibrates and omega-3 polyunsaturated fatty acids are the traditional and the most used lipid lowering therapy that reduce TG levels. An *in-vitro* study suggested that fenofibrate may reduce SARS-CoV-2 infectivity by approximately 70% through destabilizing receptor-binding domain of SARS-CoV-2 spike protein and inhibiting its binding to ACE2 (24). Another *in-vitro* study of SARS-CoV-2 in human lung epithelium suggested that peroxisome proliferator-activated receptor alpha (*PPAR-α*) activation of fatty acid oxidation using fenofibrate could disrupt the SARS-CoV-2 lifecycle by blocking phospholipid accumulation and increasing glycolysis, reversing the metabolic effects of infection. A 5-day treatment with fenofibrate reduced viral load by 2-logs (*p* < 0.001) without affecting cell viability. These results suggest that lipid metabolism is an important pathway for SARS-CoV-2 replication and a promising therapeutic target (25). Interestingly, patients in the hypertriglyceridemia treatment group showed significantly longer time to COVID-19 recovery and hospital stay compared with those in the no-treatment group. However, we propose that the patients who were in the treatment group might be sicker given that they had significantly higher temperature, levels of C-RP and IL-6, and use of invasive oxygen therapy. Moreover, significantly more patients in the treatment group received broad-spectrum antibiotics, antifungal, steroids, hydroxychloroquine, tocilizumab, interferon, and intravenous immunoglobulin. Our results of this study will be confirmed after the completion of the ongoing randomized trials that should provide more robust evidence, given the main limitation of our observational study design with its inherent limitations. Another important limitation is presenting the crude relative association measure (i.e., OR) since the significant parameters that have been tested did not show significant finding when regression analysis was performed. At least three ongoing randomized controlled trials that are examining fibrate compared with placebo in hospitalized non-ICU and outpatient COVID-19 patients (NCT04661930, NCT04517396, PER-099020) and enrolling 50 to 700 participants. Omega-3 fatty acids may have a beneficial role in COVID-19 by activating lipid mediators that decrease inflammation. There are at least six randomized trials investigating omega-3 acid products in hospitalized non-ICU patients (IRCT20200511047399N1, NCT04335032, NCT04507867, NCT04553705, NCT04647604, RBR-7jrxqm) and four in the outpatients setting (NCT04357990, NCT04460651, NCT04412018, NCT04495816), which are recruiting 30 to 284 and 30 to 4,000 participants, respectively. In addition, there are ongoing randomized trials on the use of omega-3 fatty acids products in preventing COVID-19 (NCT4505098, NCT04460651, NCT04483271, NCT04658433) which are enrolling 100 to 16,500 participants (26).

## 5 Conclusion

Hypertriglyceridemia associated with COVID-19 did not increase death rate, but prolonged time to death and length of stay which may indicate possible a more complex course of illness. Treating hypertriglyceridemia did not translate into improvement in clinical outcomes including mortality.

## Data availability statement

The datasets used and/or analyzed during the current study are available from the corresponding author on reasonable request.

## Ethics statement

The studies involving humans were approved by Medical Research Center (MRC) of Hamad Medical Corporation. The studies were conducted in accordance with the local legislation and institutional requirements. The ethics committee/institutional review board waived the requirement of written informed consent for participation from the participants or the participants' legal guardians/next of kin because retrospective design; not interventional study.

## Author contributions

RK: Conceptualization, Methodology, Supervision, Validation, Writing – original draft, Writing – review & editing. MM: Formal analysis, Software, Validation, Writing – review & editing. MA-A: Methodology, Validation, Writing – review & editing. AP: Data curation, Methodology, Validation, Writing – review & editing. AA: Data curation, Methodology, Writing – review & editing. SA: Data curation, Supervision, Writing – review & editing. MA: Conceptualization, Methodology, Validation, Writing – review & editing.

## References

[1] World Health Organization. Coronavirus Disease 19 (COVID-19)-Situation Report; (2020). Available at: https://covid19.who.int (Accessed March 11, 2020).

[2] Tang Q Gao L Tong Z Li W. Hyperlipidemia, COVID-19 and acute pancreatitis: a tale of three entities. Am J Med Sci. (2022) 364:257–63. doi: 10.1016/j.amjms.2022.03.007, PMID: 35381217 PMC8977370

[3] Kaddoura R Salam AM. Thrombosis management and challenges in COVID-19 patients presenting with acute coronary syndromes. Heart Views. (2020) 21:195–208. doi: 10.4103/HEARTVIEWS.HEARTVIEWS_143_20, PMID: 33688412 PMC7898995

[4] Omar AS Kaddoura R Orabi B Hanoura S. Impact of COVID-19 pandemic on liver, liver diseases, and liver transplantation programs in intensive care units. World J Hepatol. (2021) 13:1215–33. doi: 10.4254/wjh.v13.i10.1215, PMID: 34786163 PMC8568568

[5] Gadiparthi C Bassi M Yegneswaran B Ho S Pitchumoni CS. Hyperglycemia, hypertriglyceridemia, and acute pancreatitis in COVID-19 infection: clinical implications. Pancreas. (2020) 49:e62–3. doi: 10.1097/MPA.0000000000001595, PMID: 32604205 PMC7375186

[6] Wei X Zeng W Su J Wan H Yu X Cao X. Hypolipidemia is associated with the severity of COVID-19. J Clin Lipidol. (2020) 14:297–304. doi: 10.1016/j.jacl.2020.04.008, PMID: 32430154 PMC7192140

[7] Roccaforte V Daves M Lippi G Spreafico M Bonato C. Altered lipid profile in patients with COVID-19 infection. J Lab Precis Med. (2021) 6:2. doi: 10.21037/jlpm-20-98

[8] Pérez-Torres I Guarner-Lans V Soria-Castro E Manzano-Pech L Palacios-Chavarría A Valdez-Vázquez RR. Alteration in the lipid profile and the desaturases activity in patients with severe pneumonia by SARS-CoV-2. Front Physiol. (2021) 12:667024. doi: 10.3389/fphys.2021.667024, PMID: 34045976 PMC8144632

[9] Thomas CM Vicent M Moore S Ali F Wooten L Louzon PR. Treatment of severe hypertriglyceridemia with insulin infusions in severe COVID-19: a case series. J Pharm Pract. (2022) 35:1044–8. doi: 10.1177/08971900211010473 33882724

[10] Garg R Rustagi T. Management of Hypertriglyceridemia Induced Acute Pancreatitis. Biomed Res Int. (2018) 2018:1–12. doi: 10.1155/2018/4721357 PMC6083537 30148167

[11] World Health Organization, Clinical Management of Severe Acute Respiratory Infection (SARI) When COVID-19 Disease Is Suspected. Interim Guidance; (2020). Available at: https://www.who.int/docs/default-source/coronaviruse/clinical-management-of-novel-cov.pdf?sfvrsn=bc7da517_10&download=true (Accessed March 11, 2020).

[12] Grundy SM Stone NJ Bailey AL Beam C Birtcher KK Blumenthal RS. 2018 AHA/ACC/AACVPR/AAPA/ABC/ACPM/ADA/AGS/APhA/ASPC/NLA/PCNA guideline on the Management of Blood Cholesterol: a report of the American College of Cardiology/American Heart Association task force on clinical practice guidelines. Circulation. (2019) 139:e1082–143. doi: 10.1161/CIR.0000000000000625, PMID: 30586774 PMC7403606

[13] Bellia A Andreadi A Giudice L de Taddeo S Maiorino A D’Ippolito I. Atherogenic dyslipidemia on admission is associated with poorer outcome in people with and without diabetes hospitalized for COVID-19. Diabetes Care. (2021) 44:2149–57. doi: 10.2337/dc20-2838, PMID: 34253561

[14] Yoshikawa M Asaba K Nakayama T. Estimating causal effects of atherogenic lipid-related traits on COVID-19 susceptibility and severity using a two-sample Mendelian randomization approach. BMC Med Genet. (2021) 14:269. doi: 10.1186/s12920-021-01127-2, PMID: 34774031 PMC8590430

[15] Noor AM Farea Z Majumder S Zafar RB Balhamar A Mady AF. Hypertriglyceridemia in a COVID-19 patient: a case report. Int J Health Sci Res. (2022) 12:316–20. doi: 10.52403/ijhsr.20220437

[16] Fijen LM Grefhorst A Levels JHM Cohn DM. Severe acquired hypertriglyceridemia following COVID-19. BMJ Case Rep. (2021) 14:e246698. doi: 10.1136/bcr-2021-246698, PMID: 34764129 PMC8587683

[17] Cetinkaya A Erden A Avci D Karagoz H Karahan S Basak M. Is hypertriglyceridemia a prognostic factor in sepsis? Ther Clin Risk Manag. (2014) 10:147–50. doi: 10.2147/TCRM.S57791 24600230 PMC3942219

[18] Feingold KR Staprans I Memon RA Moser AH Shigenaga JK Doerrler W. Endotoxin rapidly induces changes in lipid metabolism that produce hypertriglyceridemia: low doses stimulate hepatic triglyceride production while high doses inhibit clearance. J Lipid Res. (1992) 33:1765–76. doi: 10.1016/S0022-2275(20)41334-3, PMID: 1479286

[19] Feingold KR Grunfeld C. Tumor necrosis factor-alpha stimulates hepatic lipogenesis in the rat in vivo. J Clin Invest. (1987) 80:184–90. doi: 10.1172/JCI113046 3597772 PMC442217

[20] Grunfeld C Gulli R Moser AH Gavin LA Feingold KR. Effect of tumor necrosis factor administration in vivo on lipoprotein lipase activity in various tissues of the rat. J Lipid Res. (1989) 30:579–85. doi: 10.1016/S0022-2275(20)38349-8, PMID: 2754338

[21] Semb H Peterson J Tavernier J Olivecrona T. Multiple effects of tumor necrosis factor on lipoprotein lipase in vivo. J Biol Chem. (1987) 262:8390–4. doi: 10.1016/S0021-9258(18)47576-X, PMID: 3597377

[22] Morrison AR Johnson JM Ramesh M Bradley P Jennings J Smith ZR. Acute hypertriglyceridemia in patients with COVID-19 receiving tocilizumab. J Med Virol. (2020) 92:1791–2. doi: 10.1002/jmv.25907, PMID: 32314799 PMC7264729

[23] Jalal Eldin A Lyonga A Ohiokpehai B Rizwan M Musa A. COVID-19, hypertriglyceridemia, and acute pancreatitis: a case report and clinical considerations. Cureus. (2023) 15:e35431. doi: 10.7759/cureus.35431, PMID: 36994303 PMC10040487

[24] Davies SP Mycroft-West CJ Pagani I Hill HJ Chen YH Karlsson R. The Hyperlipidaemic drug Fenofibrate significantly reduces infection by SARS-CoV-2 in cell culture models. Front Pharmacol. (2021) 12:660490. doi: 10.3389/fphar.2021.660490, PMID: 34421587 PMC8377159

[25] Ehrlich A Uhl S Ioannidis K Hofree M TenOever BR Nahmias Y. The SARS-CoV-2 transcriptional metabolic signature in lung epithelium. SSRN. (2020):3650499. doi: 10.2139/ssrn.3650499

[26] Talasaz AH Sadeghipour P Aghakouchakzadeh M Dreyfus I Kakavand H Ariannejad H. Investigating lipid-modulating agents for prevention or treatment of COVID-19: JACC state-of-the-art review. J Am Coll Cardiol. (2021) 78:1635–54. doi: 10.1016/j.jacc.2021.08.021, PMID: 34649702 PMC8504484

