# Supplementary material for: COVID-19-associated hypertriglyceridemia and impact of treatment
Source: Front Med (Lausanne). 2024 Feb 21;11:1326156. doi: 10.3389/fmed.2024.1326156 (PMC10915025; doi:10.3389/fmed.2024.1326156)
Supplement: Supplementary file 1 [file Table_1.docx]

**Supplementary tables**

**Table S1**. Vital signs and laboratory blood tests based on triglycerides levels

| Variable | High triglycerides levels | | p value |
| --- | --- | --- | --- |
|  | Yes (median, Δ IQR)  N=1016 | No (median, Δ IQR)  N=218 |  |
| Vital signs and oxygen | | | |
| Temperature (^0^C) | 37.0 (0.7) | 36.8 (0.4) | 0.003 |
| Hearts rate (bpm) | 89.0 (24.0) | 82.0 (23.0) | < 0.001 |
| Respiratory rate (bpm) | 27.0 (10.0) | 24.0 (9.0) | < 0.001 |
| Mean arterial pressure, mmHg | 92.0 (18.0) | 92.2 (15.7) | 0.757 |
| SpO_2_  Oxygen saturation | 95.0 (6.0) | 96.5 (5.0) | < 0.001 |
| Oxygen flow rate (L/min) | 15.0 (5.0) | 10.0 (11.0) | < 0.001 |
| Blood tests | | | |
| WBC (x10^3^/uL) | 8.8 (5.0) | 8.8 (5.0) | 0.244 |
| Hemoglobin (g/dL) | 12.8 (3.0) | 12.7 (3.0) | 0.205 |
| Platelet (x10^3^/uL) | 256.0 (122.0) | 251.0 (146.0) | 0.249 |
| ANC (x10^3^/uL) | 7.5 (5.0) | 7.0 (6.0) | 0.020 |
| Lymphocyte (x10^3^/uL) | 0.8 (1.0) | 0.9 (1.0) | 0.003 |
| INR | 1.1 (0) | 1.1 (0) | 0.404 |
| APTT (seconds) | 31.4 (6.0) | 30.1 (7.0) | 0.347 |
| Fibrinogen (g/L) | 6.2 (2.0) | 5.8 (3.0) | < 0.001 |
| D-dimer | 1.1 (2.0) | 1.1 (6.0) | 0.242 |
| ESR (mm/h) | 41.0 (24.0) | 39.5 (23.0) | 0.260 |
| BUN (mmol/L) | 5.9 (5.0) | 5.3 (4.0) | 0.021 |
| Creatinine (umol/L) | 81.0 (34.0) | 69.5 (35.0) | 0.029 |
| ALT (U/L) | 40.0 (38.0) | 34.0 (35.0) | 0.003 |
| AST (U/L) | 48.0 (43.0) | 43.0 (36.0) | 0.003 |
| Total bilirubin (umol/L) | 10.0 (7.0) | 10.0 (7.0) | 0.745 |
| Albumin (g/L) | 27.0 (6.0) | 30.0 (8.0) | < 0.001 |
| Glucose (mmol/L) | 9.4 (9.0) | 8.6 (6.0) | 0.009 |
| LDH (U/L) | 510.0 (241.0) | 445.5 (210.0) | < 0.001 |
| C-RP (mg/L) | 145.8 (143.0) | 88.0 (123.0) | < 0.001 |
| Procalcitonin (ng/mL) | 0.4 (1.0) | 0.2 (1.0) | 0.016 |
| Ferritin (ug/L) | 1268.2 (1076.0) | 620.0 (757.0) | < 0.001 |
| IL-6 (pg/mL) | 79.0 (203.0) | 48.0 (111.0) | 0.007 |
| Lactate (mmol/L) | 1.7 (1.0) | 1.5 (2.0) | 0.436 |
| *Abbreviations: ALT, alanine transaminase; ANC, absolute neutrophil count; APTT, activated partial thromboplastin; AST, aspartate aminotransferase; bpm, beats or breaths per minute; BUN, blood urea nitrogen; C-RP, C-reactive protein; ESR, erythrocyte sedimentation rate; IL-6, interleukin-6; INR, international normalized ratio; IQR, interquartile range; LDH, lactate dehydrogenase; WBC, white blood cells.* | | | |

**Table S2**. Triglyceride level values during Admission

| Variable (mmol/L) | High triglycerides levels | | | |
| --- | --- | --- | --- | --- |
|  | Yes | | No | |
|  | Mean (SD) | Median (Δ IQR) | Mean (SD) | Median (Δ IQR) |
| Baseline TG | 2.8 (1.7) | 2.4 (1.5) | 1.2 (0.5) | 1.1 (0.5) |
| TG before ICU | 2.8 (1.8) | 2.4 (1.6) | 1.1 (0.3) | 1.1 (0.6) |
| TG in ICU | 2.9 (1.7) | 2.4 (1.5) | 1.2 (0.4) | 1.1 (0.5) |
| Highest TG in ICU | 4.3 (2.2) | 3.7 (2.8) | 1.3 (0.3) | 1.3 (0.4) |
| Lowest TG in ICU | 1.8 (0.9) | 1.0 (0.4) | 1.0 (0.3) | 1.0 (0.4) |
| *Abbreviations: ICU, intensive care unit; IQR, interquartile range; SD, standard deviation; TG, triglyceride.* | | | | |

**Table S3**. Clinical outcomes based on triglycerides levels

| Variable | High triglycerides levels | | p value |
| --- | --- | --- | --- |
|  | Yes (median, Δ IQR)  N=1016 | No (median, Δ IQR)  N=218 |  |
| Time to COVID-19 recovery (day) | 21.5 (14.9) | 16.3 (14.6) | 0.003 |
| Length of ICU stay (day) | 10.1 (14.0) | 5.7 (10.0) | < 0.001 |
| Length of hospital stay (day) | 24.5 (17.7) | 20.3 (14.6) | < 0.001 |
| Time between death and first positive PCR (day) | 29.3 (31.8) | 16.9 (17.0) | < 0.001 |
|  | (n, %) | (n, %) |  |
| Death (within 30 days of diagnosis) | 98 (9.6) | 28 (12.8) | 0.157 |
| Death (within 60 days of diagnosis) | 159 (15.6) | 33 (15.1) | 0.850 |
| *Abbreviations: ICU, intensive care unit; IQR, interquartile range; PCR, polymerase chain reaction.* | | | |

**Table S4**. Baseline characteristics based on hypertriglyceridemia treatment

| Variable | Hypertriglyceridemia treatment | | p value |
| --- | --- | --- | --- |
|  | Yes (n, %)  N=343 (33.8%) | No (n, %)  N=673 (66.2%) |  |
| Male  Female | 311 (90.7)  32 (9.3) | 582 (86.5)  91 (13.5) | 0.053 |
| Age group (years)  ≤ 20  21-30  31-40  41-50  51-60  > 60 | 0 (0)  16 (4.7)  76 (22.2)  117 (34.1)  78 (22.7)  56 (16.3) | 2 (0.3)  28 (4.2)  111 (16.5)  184 (27.3)  191 (28.4)  157 (23.3) | 0.005 |
| Qatari  Non-Qatari | 25 (7.3)  318 (92.7) | 47 (7.0)  626 (93.0) | 0.858 |
| Age (year), median (Δ IQR) | 47.5 (14.0) | 52.0 (18.0) | < 0.001 |
| BMI (kg/m^2^), median (Δ IQR) | 27.8 (6.9) | 28.0 (6.8) | 0.367 |
| Smoking and alcohol status | | | |
| Non-smoker  Smoker  Ex-smoker  Unknown | 186 (54.2)  15 (4.4)  15 (4.4)  127 (37.0) | 376 (55.9)  32 (4.8)  39 (5.8)  226 (33.5) | 0.401 |
| Non-alcoholic  Alcoholic  Former  Unknown | 164 (47.8)  18 (5.2)  6 (1.7)  155 (45.3) | 354 (52.5)  21 (31.2)  17 (2.5)  281 (41.8) | 0.772 |
| Comorbidities | | | |
| Hypertension | 158 (46.1) | 372 (55.3) | 0.005 |
| Diabetes mellitus | 191 (55.7) | 410 (60.9) | 0.108 |
| CAD | 26 (7.6) | 62 (9.2) | 0.382 |
| PVD | 3 (0.9) | 2 (0.3) | 0.214 |
| History of DVT/PE | 15 (4.4) | 46 (6.8) | 0.118 |
| Heart Failure | 15 (4.4) | 40 (5.9) | 0.296 |
| Atrial fibrillation | 23 (6.7) | 66 (9.8) | 0.098 |
| CKD/ESRD | 38 (11.1) | 105 (15.6) | 0.050 |
| Liver disease | 15 (4.4) | 41 (6.1) | 0.256 |
| COPD | 7 (2.0) | 18 (2.7) | 0.537 |
| ILD | 1 (0.3) | 4 (0.6) | 0.668 |
| PUD | 2 (0.6) | 10 (1.5) | 0.357 |
| Solid tumor | 6 (1.8) | 21 (3.1) | 0.199 |
| Lymphoma | 3 (0.9) | 4 (0.6) | 0.694 |
| Leukemia | 2 (0.6) | 8 (1.2) | 0.509 |
| Organ transplant | 8 (2.3) | 8 (1.2) | 0.166 |
| Dementia | 2 (0.6) | 11 (1.6) | 0.239 |
| Note: Chi-Square, Fisher Exact’s, and Mann-Whitney tests were used at alpha 0.05  *Abbreviations: CAD, coronary artery disease; CKD, chronic kidney disease; COPD, chronic obstructive pulmonary disease; DVT, deep vein thrombosis; ESRD, end-stage renal disease; ILD, interstitial lung disease; IQR, interquartile range; PE, pulmonary embolism; PUD, peptic ulcer disease; PVD, peripheral vascular disease.* | | | |

**Table S5**. Vital signs and laboratory blood tests based on hypertriglyceridemia treatment

| Variable | Hypertriglyceridemia treatment | | p value |
| --- | --- | --- | --- |
|  | Yes (median, Δ IQR)  N=343 | No (median, Δ IQR)  N=673 |  |
| Vital signs | | | |
| Temperature (^0^C) | 37.0 (0.8) | 36.8 (0.6) | < 0.001 |
| Hearts rate (bpm) | 90.0 (23.0) | 89.0 (24.0) | 0.578 |
| Respiratory rate | 27.0 (10.0) | 28.0 (10.0) | 0.773 |
| MAP (mmHg) | 93.0 (18.0) | 91.7 (17.8) | 0.346 |
| SpO_2_ | 95.0 (5.0) | 95.0 (6.0) | 0.633 |
| O_2_ flow rate (L/min) | 12.0 (5.0) | 15.0 (5.0) | 0.022 |
| Blood tests | | | |
| WBC (x10^3^/uL) | 8.6 (5.0) | 8.9 (6.0) | 0.444 |
| Hemoglobin (g/dL) | 12.6 (2.0) | 12.9 (3.0) | 0.415 |
| Platelet (x10^3^/uL) | 254.0 (120.0) | 254.0 (124.0) | 0.710 |
| ANC (x10^3^/uL) | 7.2 (5.0) | 7.7 (6.0) | 0.407 |
| Lymphocyte (x10^3^/uL) | 0.8 (1.0) | 0.8 (1.0) | 0.349 |
| INR | 1.1 (0) | 1.1 (0) | 0.519 |
| APTT (seconds) | 31.4 (6.0) | 31.2 (6.0) | 0.599 |
| Fibrinogen (g/L) | 5.8 (3.0) | 6.0 (2.0) | 0.393 |
| D-Dimer | 1.1 (2.0) | 1.1 (3.0) | 0.749 |
| ESR (mm/h) | 41.0 (23.0) | 41.0 (25.0) | 0.083 |
| BUN (mmol/L) | 5.5 (4.0) | 6.5 (5.0) | 0.027 |
| Creatinine (umol/L) | 83.5 (33.0) | 80.0 (34.0) | 0.034 |
| ALT (U/L) | 42.5 (36.0) | 43.0 (37.0) | 0.781 |
| AST (U/L) | 50.5 (42.0) | 52.0 (45.0) | 0.740 |
| Total bilirubin (umol/L) | 10.0 (8.0) | 10.0 (7.0) | 0.047 |
| Albumin (g/L) | 27.0 (6.0) | 26.0 (6.0) | 0.538 |
| Glucose (mmol/L) | 9.7 (11.0) | 8.9 (7.0) | 0.133 |
| LDH (U/L) | 539.0 (247.0) | 551.0 (229.0) | 0.410 |
| C-RP (mg/L) | 163.6 (82.0) | 137.2 (140.0) | **0.013** |
| Procalcitonin (ng/mL) | 0.4 (1.0) | 0.5 (1.0) | 0.256 |
| Ferritin (ug/L) | 1363.0 (1093.0) | 971.0 (1361.0) | 0.319 |
| IL-6 (pg/mL) | 159.0 (277.0) | 137.5 (303.0) | **0.020** |
| Lactate (mmol/L) | 1.8 (1.0) | 1.7 (1.0) | 0.449 |
| *Abbreviations: ALT, alanine transaminase; ANC, absolute neutrophil count; APTT, activated partial thromboplastin; AST, aspartate aminotransferase; bpm, beats or breaths per minute; BUN, blood urea nitrogen; C-RP, C-reactive protein; ESR, erythrocyte sedimentation rate; IL-6, interleukin-6; INR, international normalized ratio; IQR, interquartile range; LDH, lactate dehydrogenase; WBC, white blood cells.* | | | |

**Table S6**. Triglyceride level values based on hypertriglyceridemia treatment

| Variable (mmol/L) | Hypertriglyceridemia treatment | | | |
| --- | --- | --- | --- | --- |
|  | Yes | | No | |
|  | Mean (SD) | Median (Δ IQR) | Mean (SD) | Median (Δ IQR) |
| Baseline TG | 3.2 (2.0) | 2.5 (2.1) | 2.8 (1.9) | 2.4 (1.6) |
| TG before ICU | 3.1 (1.8) | 2.5 (1.9) | 2.8 (1.9) | 2.4 (1.6) |
| Highest TG in ICU | 3.2 (1.9) | 2.6 (2.1) | 2.9 (2.0) | 2.4 (1.6) |
| Lowest TG in ICU | 2.0 (1.5) | 1.6 (1.2) | 1.6 (0.8) | 1.4 (0.8) |
| *Abbreviations: ICU, intensive care unit; IQR, interquartile range; SD, standard deviation; TG, triglyceride.* | | | | |

**Table S7**. At-home and in-hospital medications based on hypertriglyceridemia treatment

| Variable | Hypertriglyceridemia treatment | | p value |
| --- | --- | --- | --- |
|  | Yes (n, %)  N=343 | No (n, %)  N=673 |  |
| Pre-specified medications at home | | | |
| Olanzapine | 1 (0.3) | 3 (0.5) | 1.000 |
| Cyclosporine | 1 (0.3) | 3 (0.5) | 1.000 |
| Fenofibrate | 9 (2.6) | 11 (1.6) | 0.283 |
| Gemfibrozil | 2 (0.6) | 1 (0.1) | 0.265 |
| Methylprednisolone | 11 (3.2) | 36 (5.3) | 0.124 |
| Prednisolone | 23 (6.7) | 49 (7.3) | 0.735 |
| Tacrolimus | 4 (1.2) | 4 (0.6) | 0.454 |
| Hypertriglyceridemia treatment in hospital | | | |
| Gemfibrozil | 8 (2.3) | - | - |
| Omega-3 | 125 (36.4) | - | - |
| Omega-3 and fenofibrate | 39 (11.4) | - | - |
| Fenofibrate | 253 (73.8) | - | - |
| Medications in hospital | | | |
| Insulin | 321 (93.6) | 592 (88.0) | 0.018 |
| Olanzapine | 2 (0.6) | 4 (0.6) | 0.135 |
| Atorvastatin | 50 (14.8) | 142 (21.1) | 0.003 |
| Pravastatin | 0 (0) | 3 (0.5) | 0.061 |
| Rosuvastatin | 27 (7.9) | 54 (8.0) | 0.132 |
| Simvastatin | 2 (0.6) | 4 (0.6) | 0.135 |
| Any statin | 74 (21.8) | 186 (27.6) | 0.015 |
|  | | | |
| Beta-lactams* | 327 (95.3) | 614 (91.2) | 0.018 |
| Meropenem/piperacillin-tazobactam | 287 (83.7) | 470 (69.8) | <0.001 |
| Antifungal agents^&^ | 132 (38.5) | 140 (20.8) | <0.001 |
| Hydroxychloroquine | 308 (89.8) | 527 (78.3) | <0.001 |
| Azithromycin | 329 (95.9) | 618 (91.8) | 0.014 |
| Methylprednisolone/dexamethasone | 325 (94.8) | 601 (89.3) | 0.004 |
| Tocilizumab | 249 (72.6) | 367 (54.5) | <0.001 |
| Interferon | 35 (10.2) | 105 (15.6) | 0.018 |
| IVIG use | 109 (31.8) | 105 (15.6) | <0.001 |
| Oseltamivir | 329 (95.9) | 618 (91.8) | 0.014 |
| Lopinavir/ritonavir | 213 (62.1) | 349 (51.9) | 0.002 |
| Favipiravir | 40 (11.7) | 134 (19.9) | <0.001 |
| Darunavir | 15 (4.4) | 28 (4.2) | 0.873 |
| Ribavirin | 35 (10.2) | 86 (12.8) | 0.231 |
| VTE prophylaxis^#^ | 340 (99.1) | 668 (99.3) | 1.000 |
| Oxygen therapy | | | |
| Non-invasive O_2_ therapy | 338 (98.5) | 658 (97.8) | 0.403 |
| Invasive O_2_ therapy | 253 (73.8) | 406 (60.3) | <0.001 |
| All O_2_ Therapy | 338 (98.5) | 659 (97.9) | 0.489 |
|  | Median (Δ IQR) | Median (Δ IQR) |  |
| Duration of non-invasive O_2_ (day) | 16.2 (19.7) | 16.9 (14.0) | 0.625 |
| Duration of invasive O_2_ (day) | 5.6 (13.0) | 3.1 (16.5) | 0.001 |
| Duration of all O_2_ (Day) | 23.4 (22.5) | 21.9 (26.5) | 0.169 |
| Note: Chi-Square test and Fisher Exact’s test were used at alpha 0.05  *Ceftriaxone, co-amoxiclav, cefepime, ceftazidime  &Anidulafungin, caspofungin, amphotericin  #Heparin, enoxaparin, fondaparinux, or dalteparin  *Abbreviations: IQR, interquartile range; IVIG, intravenous immunoglobulin; O_2_, oxygen; VTE, venous thromboembolism.* | | | |

**Table S8**. Clinical outcomes based on hypertriglyceridemia treatment

| Variable | Hypertriglyceridemia treatment | | p value |
| --- | --- | --- | --- |
|  | Yes, (median, Δ IQR)  N=343 | No, (median, Δ IQR)  N=673 |  |
| Time to COVID-19 recovery (day) | 24.4 (21.0) | 18.7 (14.4) | <0.001 |
| Length of ICU stay (day) | 11.4 (17.0) | 12.4 (14.0) | 0.671 |
| Length of hospital stay (day) | 30.4 (15.4) | 23.9 (21.9) | <0.001 |
| Time between death and first positive PCR (day) | 40.0 (28.7) | 27.2 (32.0) | 0.020 |
|  | (n, %) | (n, %) |  |
| Death (Within 30 days of Diagnosis) | 31(9.0) | 67 (10.0) | 0.639 |
| Death (Within 60 days of Diagnosis) | 60 (17.5) | 99 (14.7) | 0.248 |
| *Abbreviations: ICU, intensive care unit; IQR, interquartile range; PCR, polymerase chain reaction.* | | | |
